# Toolkit for Monitoring and Evaluation of Indoor Residual Spraying for Visceral Leishmaniasis Control in the Indian Subcontinent: Application and Results

**DOI:** 10.1155/2011/876742

**Published:** 2011-07-27

**Authors:** M. Mamun Huda, Dinesh Mondal, Vijay Kumar, Pradeep Das, S. N. Sharma, Murari Lal Das, Lolita Roy, Chitra Kumar Gurung, Megha Raj Banjara, Shireen Akhter, Narayan Prosad Maheswary, Axel Kroeger, Rajib Chowdhury

**Affiliations:** ^1^Laboratory Sciences Division, International Centre for Diarrhoeal Disease Research, Bangladesh (ICDDR,B), Dhaka 1212, Bangladesh; ^2^Division of Vector Biology & Control, Rajendra Memorial Research Institute of Medical Sciences (RMRIMS), Patna 800 007, India; ^3^National Vector Borne Disease Control Programme (NVBDCP), Director General and Health Services, New Delhi 110054, India; ^4^Department of Microbiology, BP Koirala Institute of Health Sciences (BPKIHS), Dharan 56700, Nepal; ^5^Department of Community Medicine and Family Health, Institute of Medicine, Tribhuvan University (IOM), Kathmandu 44613, Nepal; ^6^Department of Medical Entomology, National Institute of Preventive and Social Medicine (NIPSOM), Dhaka 1212, Bangladesh; ^7^Special Programme for Research and Training in Tropical Diseases, World Health Organization, 1211 Geneva, Switzerland; ^8^Intelligent Vector Control Consortium, Liverpool School of Tropical Medicine, Liverpool L35QA, UK; ^9^Regional Office for South-East Asia, World Health Organization, New Delhi 110002, India

## Abstract

*Background*. We field tested and validated a newly developed monitoring and evaluation (M&E) toolkit for indoor residual spraying to be used by the supervisors at different levels of the national kala-azar elimination programs in Bangladesh, India and Nepal. *Methods*. Methods included document analysis, in-depth interviews, direct observation of spraying squads, and entomological-chemical assessments (bioassay, susceptibility test, chemical analysis of insecticide residues on sprayed surfaces, vector density measurements at baseline, and three follow-up surveys). *Results*. We found that the documentation at district offices was fairly complete; important shortcomings included insufficient training of spraying squads and supervisors, deficient spray equipment, poor spraying performance, lack of protective clothing, limited coverage of houses resulting in low bioavailability of the insecticide on sprayed surfaces, and reduced vector susceptibility to DDT in India, which limited the impact on vector densities. *Conclusion*. The M&E toolkit is a useful instrument for detecting constraints in IRS operations and to trigger timely response.

## 1. Introduction

Visceral leishmaniasis (VL)/kala-azar (KA) is a public health problem in the Indian subcontinent, particularly in Bangladesh, India and Nepal [1]. The elimination of the disease is possible due to its unique epidemiological features including easy diagnosis at the field level with the rK39 rapid diagnostic test, availability of highly effective drugs, and effective vector control methods if applied correctly [2, 3]. In a previous study, it was highlighted that there has been no VL vector control programme in Bangladesh for a long time [4], and that indoor residual spraying (IRS) with DDT in India and lambdacyhalothrin in Nepal was effective in reducing sandfly vectors under controlled conditions but showed many shortcomings within national programmes [5, 6]. IRS is expensive and operationally a challenge; if not done properly it would be a wastage of human and financial resources. Thus systematic monitoring and evaluation of IRS activities is of paramount importance to have an impact on the vector population. A monitoring and evaluation (M&E) toolkit is highly desirable for detecting operational issues at an early stage so that they can be addressed adequately. Therefore such a toolkit has been developed in an interagency effort including the Special Programme for Tropical Disease Research and Training (TDR) at the World Health Organization (WHO) in Geneva, the German Cooperation (BMZ/GIZ), scientists from different research institutes as well as programme managers from Bangladesh, India and Nepal. The present study investigates the process and results of the application of the M&E toolkit at the supervisory level by spray supervisors and District Malaria Officers (DMOs)/District Public Health Officers (DPHOs)/Upazila Health and Family Planning Officers (UH&FPOs) with regard to identifying limiting factors of the routine national IRS activities in India and Nepal, and in two pilot areas of Bangladesh.

## 2. Methods

### 2.1. Monitoring and Evaluation Toolkit

**Figure 1:**
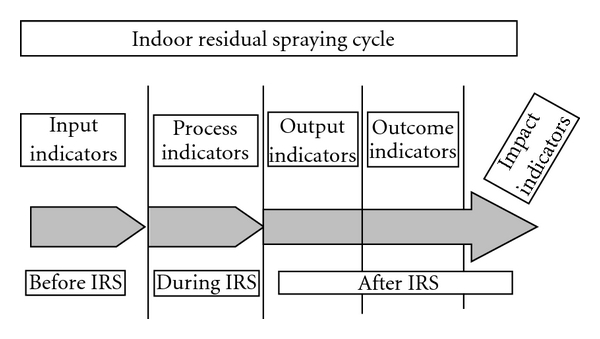
M&E toolkit for IRS.

The main purpose of the M&E toolkit is to support IRS programmes through systematic monitoring and evaluation of processes and outcomes, allowing timely detection of gaps and constraints and ensuring that adequate responses are taken. Details about the toolkit can be found in the WHO website (http://apps.who.int/tdr/svc/publications/tdr-research-publications/irs_toolkit) [7]. It is independent of the spray schedule, of the insecticide used (in our case, DDT in India, deltamethrin in Bangladesh; lambdacyhalotrin in Nepal) and of the spray pumps (compressor pumps in Nepal and Bangladesh; stirrup pumps in India). Briefly, the toolkit contains sets of indicators classified as input, process, output, outcome, and impact indicators (Figure 1). Input indicators include national guidelines and action plans for IRS, engagement and training of spray teams, availability of insecticides, spray pumps, spare parts, storage facilities, and protective materials. Process indicators include all information related to the performance of spraying and quantity of insecticide used. Output indicators include the coverage achieved by IRS through document analysis and household interviews, assessment of the bioavailability of the insecticide on sprayed surfaces through the WHO cone bioassay test, determination of the chemical concentration achieved on sprayed surfaces (filter paper method), and the level of acceptability of IRS by the community. The outcome indicator presents the effect of IRS on the sandfly density. Finally, health impact is measured as the reduction of disease burden in the community. The toolkit is complemented by a data entry programme which facilitates the data entry at district/subdistrict levels.

### 2.2. Study Design, Population, and Timeline

**Figure 2:**
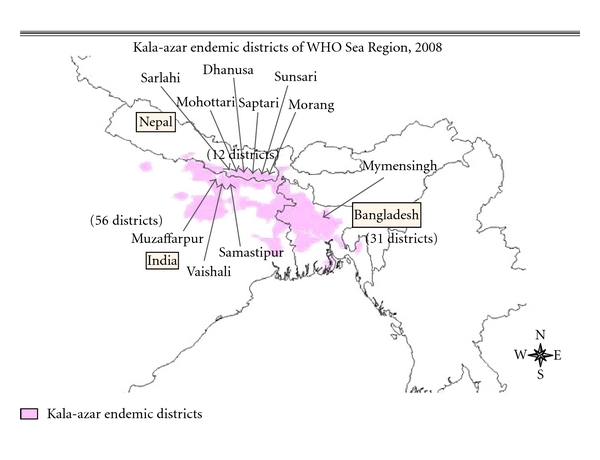
Study area map.

The study has been carried out from April 2009 to June 2010 in 9 districts in India and Nepal and in two Upazilas (subdistricts) in Bangladesh by 5 research teams: Institute of Medicine (IOM) at the Tribhuvan University in Kathmanadi/Nepal (Sarlahi, Dhanusha and Mahottari districts); BP Koirala Institute of Health Sciences (BPKIHS) in Dharan, Nepal (Morang, Sunsari, and Saptari); Rajendra Memorial Research Institute of Medical Sciences (RMRIMS) in Patna, Bihar, India (Muzaffarpur, Vaishali, and Samastipur) and International Centre for Diarrhoeal Disease Research, Bangladesh (ICDDR,B), and the National Institute of Preventive and Social Medicine (NIPSOM), Dhaka, Bangladesh (Trishal Upazilla and Fulbaria Upazila in Mymensingh District) (Figure 2). The study sites were purposively selected based on high VL endemicity.

**Figure 3:**
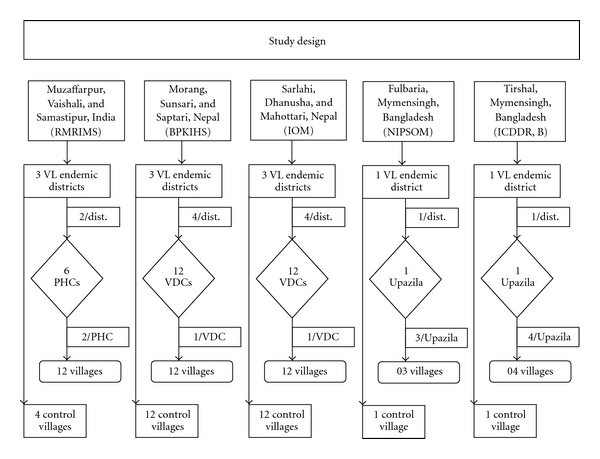
Study design.

In India, two primary health care centres (PHCs) were selected from each of the above-mentioned districts, in total six PHCs, chosen on the basis of previous VL case load. Twelve villages (two from each PHC, one close and one far away from the PHC) were selected randomly for assessing the IRS operations. Additionally, four villages were randomly selected as control area for the assessment of vector densities. In Nepal, in the six study districts, 24 village development committees (VDCs) were selected based on high VL case load according to passive surveillance data. In each district, two VDCs were near and two were distant from the district headquarter from where the IRS program was operated. The 24 VDCs were then divided in those where IRS was programmed and those where it was not. From each VDC one study village was selected randomly so that in the end 12 IRS intervention villages and 12 control villages were assessed. In Bangladesh, 3 and 4 villages were selected from Fulbaria and Trishal Upazila, respectively, according to the VL endemicity, and IRS has been carried out by the programme. Additionally, in each Upazila one village far away from the IRS village was selected randomly as a control (Figure 3). 

All vector control staff involved in IRS operations from central to field level including spray men were assessed in the study and a random selection of households in IRS areas were interviewed.

### 2.3. Sample Size Calculations

#### 2.3.1. For Vector Density Measurement

The main outcome variable of IRS is the reduction of vector density. Hence the assumptions for the sample size calculations for vector density measurement were as follows. An 80% reduction of vector density would be achieved at 4 weeks after IRS; the sandfly counts were expected to follow a negative binomial distribution with a dispersion parameter of *k* = 0.10, requiring an 80% power to detect a difference between baseline and followup at 5% significance level and assuming an intracluster coefficient at village level of ICC = 0.03. A minimal sample size of 72 households vector density measurements equally distributed over 12 villages was required. It was decided to use light traps in 12 villages and 6 households per village.

#### 2.3.2. For the Observation of Spray Performance

The sample-size calculation for analysing spraying activities was based on the assumption that 70% of the observed spraying activities would be of an acceptable standard (with 15% precision and 95% confidence level). This resulted in a target of at least 36 different spray teams to be observed in each study area. All sites (except one) exceeded the minimum number ensuring a higher power of the study.

#### 2.3.3. For the Household Acceptability Survey

Anticipating a coverage and acceptance of 80% and requiring a 95% confidence interval of the length +/−5 units, the sample size of households to be interviewed is 246. Given the inclusion of 12 villages, a sample size of 30 houses per village was found to be feasible resulting in 360 households per study site.

### 2.4. Data Collection for Input Indicators

#### 2.4.1. Interviews with Programme Personnel at Central Level

Information regarding input indicators, that is, existence of guideline and national action plan, certification of chemical in use, training of spray teams, and availability of insecticide was collected before IRS using a structured questionnaire and interviewed by the investigators from Director, National Vector Borne Disease Control Programme (NVBDCP) and Senior Programme Officer, State Health Society, Bihar, and District Malaria Officer (DMO) in India; Director, Epidemiology and Disease Control Division (EDCD) in Nepal and director, Disease control; Deputy Programme Manager (DPM), Kala-azar Control Programme and Chief Health Superintendent (CHS) of Directorate General of Health Services (DGHS) in Bangladesh.

#### 2.4.2. Interview with Programme Personnel at Peripheral Level

The detailed information regarding spraying activities (operational plan) was collected through an interview with a structured questionnaire by the investigators from Medical Officer In-Charge (MOIC), Primary Health Centre (PHC) in India and Vector Control Manager in Nepal and Upazila Health and Family Planning Officer (UH&FPO) and Health Inspector (HI) in Bangladesh.

### 2.5. Data Collection for Process Indicators

#### 2.5.1. Spray Observations

Standardized observations were made with a checklist used by trained field research assistants; the observation checklist included information about (A) mixing of insecticide, spraying techniques (width of swath, distance of nozzle from wall); management of leftover insecticide, presence of supervisor, (B) the number of houses sprayed par spray man per day and the amount of insecticide used per house were calculated in randomly selected houses.

### 2.6. Data Collection for Output Indicators

#### 2.6.1. Satisfaction and Coverage

We calculated the coverage of IRS and peoples' satisfaction through formal household interviews applied by trained field research assistants.

#### 2.6.2. Bioassays for Bioavailability of Insecticide on Sprayed Surfaces

Ten sandflies were exposed on each of 4 walls using WHO plastic cones [8], cones in 10 houses from 4 study villages. The same number was applied in unsprayed control houses. Sandflies were observed for knockdown at 1 hour and for mortality at 24 hours of recovery [9]. Corrected mortality was calculated with Abbot's formula [10] as follows:

(1) $$P=\frac{P_{i}-C}{100-C}\times 100,$$

where, *P* = corrected mortality percentage, *P*_*i*_ = percent observed mortality in insecticide exposed sandflies, *C* = percent mortality in control (nonexposed) sandflies.

Bioassays were done at 2 weeks, 4 weeks, and 5 months after IRS.

#### 2.6.3. Filter Paper Technique to Determine the Insecticide Concentration on Sprayed Walls

Before spraying and without knowledge of the spray men, four filter papers (Whatman no. 1, 5 × 5 cm^2^) were placed at different heights (from 1 foot to 6 feet from ground level) together with 9 different kinds of “fake” papers (to mislead the sprayers in case they would notice something “unusual”) on the 4 walls of each room. Filter papers were collected within a week of insecticide spraying and were air dried, coded, sealed individually in aluminum foil, and stored at 4°C until they were subjected to chemical analysis. All field-collected filter paper samples along with external standards (DDT and lambda-cyhalothrin having >95% purity) were subjected to chemical analysis by IICT (Indian Institute of Chemical Technology, Hyderabad) using standard GC—quantification methods. Fixed quantities of the test samples were injected on to high-resolution gas chromatograph in triplicate and the average values for the content of active ingredient (AI) were determined using the external standardization method.

Five DDT-WP filter papers from India and 6 lambda-cyhalothrin-WP filter papers from Nepal along with standard DDT and cyhalothrin WP formulations were retested by the WHOPES (World Health Organization Pesticide Evaluation Scheme) collaborating Centre in Belgium (Centre Wallon de Recherches Agronomiques, CRA-W, Gembloux, Belgium) and by the Liverpool School of Tropical Medicine.

#### 2.6.4. Insecticide Resistance

Insecticide susceptibility was tested in one site (India) in tube bioassays, using the WHO's standard chamber method [8]. Test papers, impregnated with 4% DDT were provided by the WHOPES collaborating centre at the School of Biological Sciences, Universiti Sains Malaysia. In each assay, 15–20 unfed, non-gravid female *P. argentipes* (caught in the study area) were introduced into a WHO susceptibility chamber lined with the insecticide-impregnated paper and left for 1 hour. In each study area, five replicates (with 15–20 and flies each) and one control, with unimpregnated paper and 20 *P. argentipes*, were run. After 1 hour of exposure, the percentage knockdown was recorded before the *P. argentipes* were taken out of the test chamber, placed in a 150 mL paper cup that was covered with netting, and maintained for 24 h at 27 ± 20 C and 80% ± 10% relative humidity, with a small cotton-wool swab soaked in 10% (w/v) sucrose solution placed on the netting top. Percentage mortality was recorded 24 h, after exposure.

### 2.7. Data Collection for Outcome Indicators

Sandfly densities were measured in IRS intervention villages and in control villages at 2-weeks before spraying and at 2, 4, and 20 weeks after spraying through the collection of sandflies with CDC light traps which is preferred to the aspirator method [11]. Additionally in India and Nepal sandfly densities were determined in sentinel houses (house in the IRS area which was not sprayed due to absence of the household members or refusal of household for spraying). In each site, 72 households where IRS was done were selected as well as 72 households which remained unsprayed (sentinel houses for determining the overall reduction of vector densities). Similarly, 72 control houses were randomly selected in unsprayed control villages next to the IRS villages. In each house one CDC light trap [11] was fixed from 6 PM to 6 AM in a standardized way in the corner of the main room 2.5–5.0 cm from the wall and 15.0 cm from the floor for one night. The captured sandflies were examined on the same day of collection. Test tubes were left in −20°C for 20 minutes or chloroform-soaked cotton was used to kill the sandflies. Identification of sandflies was based on external morphological characteristics under a binocular microscope. The sandfly species was identified, sex and abdominal conditions of females were noted separately (*P. argentipes, P. papatasi, Sergentomyia spp.*) [12].

### 2.8. Data Collection for Impact Indicators

The monitoring of VL cases over time through routine surveillance and active case detection [13] is being undertaken but is beyond the scope of this study.

### 2.9. Data Analysis

A database was developed by the data management centre at ICDDR,B in Bangladesh, using EPI Info software version 3.5.1 to enter field as well as laboratory data. Data were cleaned and checked in duplicate. Descriptive analysis was performed and 95% Confidence Interval (CI) was calculated using Normal as well as a Binomial distributional approach where applicable. The percent reduction (PR) of sandfly count attributed to IRS was calculated as:

(2) $$\text{PR}=\left[\frac{\text{IE}}{\text{mean}\;\left(A\right)}\right]*100,$$

where, IE (intervention effect) = [mean (*B*) – mean (*A*)] − [mean (*D*) − mean (*C*)], *A* = baseline value for the intervention group; *B* = postintervention value for the intervention group, *C* = baseline value for the control/sentinel group, and *D* = postintervention value for the control/sentinel group. 

The effect is negative/positive if sandfly density is decreased/increased after intervention and the effect should be zero if the sandfly density is same as baseline. All calculations were performed by STATA 10.

### 2.10. Ethical Approval

The study protocol was approved by the ethical review committee (ERC) at WHO Geneva and in each research institution by their respective ethical boards. Informed written consent was obtained from vector control staff involved with IRS operations from central to field level operation and from households which have been interviewed.

## 3. Results

### 3.1. National Control Programme Inputs into IRS

Input indicators of the M&E toolkit revealed that district action plans and guidelines for IRS were in place across the all study sites. Except for the subdistricts in Bangladesh, all other study districts in India and Nepal had proper storage facilities for insecticides. However, the proportion of functional pumps was low and the availability of spare parts was inadequate in India and in half of the study districts in Nepal (Table 1). The training of the spray men before spraying was not systematically done in India. Personal protection of the spray men was completely or partially ignored by the program in all study districts.

### 3.2. The IRS Process: Prospects and Limitations of Spraying

In general process indicators of the M&E toolkit reflected nonhomogeneous spraying performance within and between countries (Table 2). It was found in India that none of the spray squads adequately filled the pump whereas in Bangladesh and Nepal it was adequately done. In India, only 29.4% (95% CI, 16.9–41.9) of the spray squads mixed DDT and water properly while in Bangladesh and Nepal this was done properly with pyrethroids by all squads. In India during spraying, 23.5% (95% CI, 11.9–35.2) of the stirrup pumps were found to have leakages. In three study districts of Nepal and in the Trishal Upazila in Bangladesh, none of the pumps had a pressure gauze. In further three districts in Nepal, only 44.6% of pumps (95% CI, 35.4–53.9) had pressure gauzes. Most of the sites showed that proper distance of the nozzle from the surface was not maintained correctly (Table 2). In India, 41.2% (95% CI, 27.7–54.7) of spray squads did not maintain the spray swath properly. In three districts of Nepal, none of the spray squads maintained the spray swath properly whereas in the other three districts the spray squads did perfectly well. In Bangladesh, the squads in one Upazila maintained the spray swath poorly but in the other Upazila the spray squads did well. In one Upazila of Bangladesh and in India, 100% of the supervisors were present and supported the squads, but in the other Upazila of Bangladesh, less than half of the supervisors (44.2%; 95% CI, 35.3–53.1) were present. None of the supervisors at any district in Nepal were present during the spraying. The management of left over insecticides was properly done except for one Upazila in Bangladesh where most of the left over insecticide (90%; 95% CI, 84.6–95.4) was thrown in a nearby water body. The marking of sprayed houses (stencils) was partially done in all sites except in one Nepali site. Instructions had been given to households in all study sites to prepare the rooms before spraying and not to enter the house during spraying.

### 3.3. Direct Results (Outputs) of the Spraying Programme

**(a) Household Coverage**

When analyzing the records of spraying squads it was recorded that the reporting of spray activities at the operation level was very good in all study areas: 95% to 100% of target houses and target populations had been covered. Only the Indian records showed a more realistic picture: the coverage of houses and target populations was only 63.9% (95% CI, 63.6–64.2) and 64.9% (95% CI, 64.8–65.1), respectively (Table 3).

**(b) Acceptability of IRS**

IRS was highly accepted by the community people in all sites and negligible side effects were reported after IRS. However, in all study sites (except one site in Nepal) instructions from spray men were rarely received about how to prepare their houses for the spraying or to leave the house while the spraying was done or not to mud plaster or paint the wall after spraying (Table 4).

**(c) Bioavailability of the Insecticide (Bioassay Tests)**

**Figure 4:**
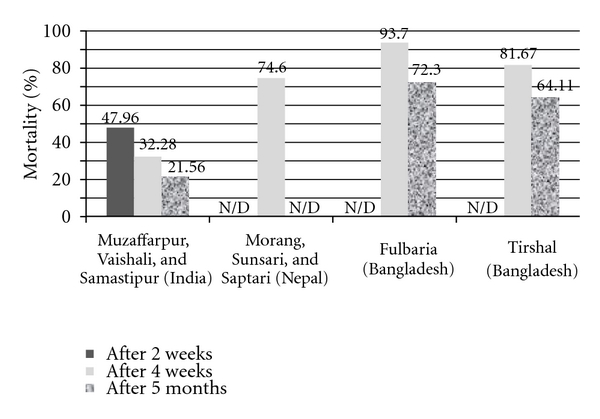
Findings of output indicator: bioassay test on IRS surfaces and bioassay results from Baijanathpur-8 and Sundarpur-3 after two weeks of spraying and from Baijanathpur-8 after four weeks of spraying were not included due to high mortality in control tests.

The bioviability of insecticides on sprayed surfaces was in all sites below the accepted standard at almost all the follow-up timepoints (Figure 4).

**(d) The Analysis of the Insecticide Concentration Reached on Sprayed Surfaces**

The test results of 311 filter papers showed that average chemical concentrations on sprayed surfaces were adequate to low in three sites (Fulbaria/Bangladesh with 15.1 mg/m^2^ (+/−14.1 SD) deltamethrin and 15.6 mg/m^2^ (+/−20.4 SD) lambdacyhalotrin in the two Nepali sites. In the “experimental area” of Trishal (pilot spraying for the national programme) there was a tendency for “overspraying” with 155.7 mg/m^2^ (+/−270.2 SD) deltamethrin. The proportion of clearly undersprayed walls (i.e., below 7 mg/m^2^) was 41.7% (90/216). Unfortunately, the results of DDT testing in filter papers by the national laboratory in India could not be reconfirmed by the laboratories in the UK and Belgium and had to be discarded.

**(e) Insecticide Resistance**

**Figure 5:**
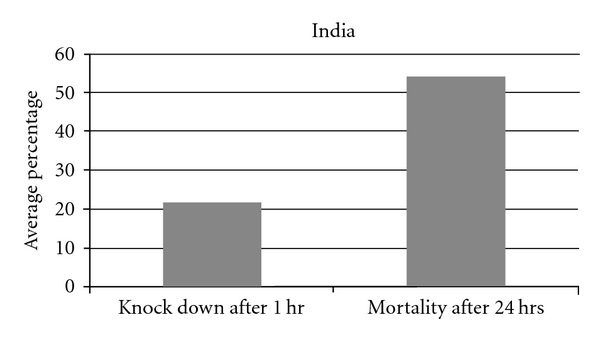
Susceptibility test for DDT in India.

Forty-four sets susceptibility test for DDT (4%) against *P. argentipes* in India have been carried out before IRS and the average sandfly knockdown rate at 1 hour was 21.5% (95% CI, 16.7–26.5), and mortality at 24 hours was 54.0% (95% CI, 48.7–59.3) (Figure 5).

**(f) Reduction of Vector Populations**

The main outcome indicator in the M&E toolkit explored whether the current national IRS programmes did achieve a high reduction rate of sandfly densities (Table 5). In India the reduction of sandflies in sprayed houses was remarkable. In Nepal IRS was effective to reduce sandfly density; however, the duration of the effect was only up to 4 weeks. In Bangladesh there was no effect on the vector population at all in one Upazila and a small effect in the other (Table 5).

## 4. Discussion

The study showed that the newly developed M&E toolkit successfully identified the shortfalls of IRS in the Indian subcontinent. IRS is an operationally challenging programme which includes a set of activities requiring (a) close coordination among national, state (India), district, and subdistrict authorities, (b) a number of technical ingredients such as insecticides, storage facilities, spraying pumps, spare parts, and protective clothing, and (c) training and supervision in order to achieve the necessary level of reduction of vector densities to interrupt disease transmission. Our study reconfirmed the original hypothesis that through systematic monitoring, the weak parts in the chain from the procurement of materials to the final endpoint reduction of sandfly vectors can be identified and solutions can be sought. The newly developed M&E toolkit was applied by spray supervisors, DMOs/DPHOs/UH&FPOs, and some elements by external reviewers from the research institutions (mainly observation of spraying performance, satisfaction survey and entomological tests). The toolkit was able to identify:

- cross-cutting issues in all or most sites: poor spraying techniques, lack of protective clothing, poor marking of houses, deficient advice to household members regarding the spraying, and limited effect on the vector population.
- site-specific problems: lack of training (Bangladesh, India), many deficient spraying pumps (India), poor filling of pumps (India), poor disposal of left-over insecticides (Bangladesh), poor marking of sprayed houses (Nepal), low coverage of targeted houses (India), high level of insecticide resistance (India).

As a result there were serious IRS effectiveness issues:

- bioassays indicated low standard of spraying and/or elevated insecticide resistance (as wild caught sandflies were used for the tests) or poor insecticide quality;
- the reduced insecticide susceptibility of sandflies for DDT in India (in contrast, in Nepal the susceptibility of sandfly vectors is still high for deltamethrin [14];
- the reduction of vector densities was low, even in India where sprayed house showed a high vector reduction but unsprayed sentinel houses showed no reduction at all pointing to a lack of mass effect on the vector population by IRS.

The chemical concentration of insecticides on sprayed surfaces as determined by a national laboratory was unexpectedly low compared to two European laboratories used for validation and quality control. These tests in the laboratory are time consuming, expensive, and susceptible to errors. Thus RDTs (rapid diagnostic test) for chemical residuals on sprayed walls are very important in this situation, and it is hoped that an RDT for chemical residuals on sprayed walls will soon be available [15].

Monitoring and evaluation activities only make sense if the findings are certified by the relevant authority and followed by adequate response mechanisms. However, this was beyond the scope of this particular study which assessed the performance of the M&E toolkit. The recommendations coming out of the analysis were summarized after completing the spraying cycle and were then discussed in training workshops with UH&FPOs in Bangladesh (2 UH&FPOs), DMOs in India (31 DMOs), and DPHOs in Nepal (16 DPHOs). These workshops were used for analyzing the acceptance of the toolkit and provided opportunities for the “triangulation” of the study results. It was found that DMOs/DPHOs/UH&FPOs were almost unanimously satisfied with the toolkit; they were aware of several issues detected in this study (e.g., inadequate training for spray men and supervisors, shortage of good quality pumps and spare parts, lack of proper supervision during spraying, etc.) but not of others (e.g., limited impact on the vector population; resistance issues) and, more importantly, further issues were raised such as the slow cash flow within districts to spraying squads resulting in delays of payment or the lack of travel funds for supervisors and sometimes even for the spray men. Meetings with the relevant district authorities were arranged in order to address these problems. It can be expected, however, that it will take some time until improved training and supervision together with the systematic application of the M&E toolkit by programme managers will be in place leading to improved IRS operations in our study districts and beyond. Of special concern is the stirrup pump used traditionally in India for DDT spraying as it is difficult to manage, less efficient than the compressor pumps used in Nepal and Bangladesh, and has higher health hazards [16]. After this study the toolkit also has been distributed by the health authorities to all districts in local language in India and Nepal, in Bangladesh translation into local language is under process.

## 5. Conclusion

The M&E toolkit was useful for identifying major shortcomings in IRS operations. Therefore the M&E toolkit developed with the help of WHO-TDR should be adopted and systematically applied by the programme of the three countries to achieve the expected outcome of IRS.

## Figures and Tables

**Table 1:** Input indicators for IRS: availability of different resources for conducting IRS.

	Samastipur, Muzaffarpur, and Vaishali (India) *N* = 3	Sunsari, Morang, and Saptari (Nepal) *N* = 3	Sarlahi, Dhanusa, and Mahottari (Nepal) *N* = 3	Fulbaria (Bangladesh)* *N* = 1	Trishal (Bangladesh)* *N* = 1
Existence of guideline and action plan	Yes	Yes	Yes	Yes	Yes
Type of insecticide to be used	DDT	Lambdacyhalothrin	Lambdacyhalothrin	Deltamethrin	Deltamethrin
Adequate storage facility	Yes	Yes	Yes	No	No
Average % of functional pumps	79.6%	94.4%	78.3%	100%	100%
Have enough spare parts	No	No	Yes	Yes	Yes
Training (days) for spray man before going to IRS	No (0 day)	Yes (3 days)	Yes (3 days)	Yes (2 days)	Yes (1 day)
Protective clothing available	Not provided	Partially provided	Partially provided	Partially provided	Partially provided

*Pilot areas for new national programme. Pumps were either hired from neighbouring sub-districts or provided by research team.

**Table 2:** Findings of process indicators: observation of spraying squad at community level.

Statements	Samastipur, Muzaffarpur, Vaishali (India)	Sunsari, Morang, Saptari (Nepal)	Sarlahi, Dhanusa, Mahottari (Nepal)	Fulbaria (Bangladesh)	Trishal (Bangladesh)
	*n* (%)	*n* (%)	*n* (%)	*n* (%)	*n* (%)
Number of spraying squads observed	51	31	112	120	120
General condition of the pump					
(i) no leakage present	39 (76.5%)	31 (100%)	112 (100%)	120 (100%)	120 (100%)
(ii) pressure gauze present	Not applicable	0 (0.00%)	50 (44.6%)	120 (100%)	0 (0.00%)
Adequate filling of the pump	0 (0.00%)	31 (100%)	112 (100%)	120 (100%)	120 (100%)
Proper mixing insecticide was done	15 (29.4%)	31 (100%)	111 (99.1%)	120 (100%)	120 (100%)
Proper distance of nozzle from surface maintained (ideally 45 cm from the surface)	25 (49.0%)	0 (0.00%)	112 (100%)	97 (80.8%)	77 (64.2%)
Proper spray swath (ideal width 65–70 cm)	30 (58.8%)	0 (0.00%)	112 (100%)	120 (100%)	78 (31.7%)
Marking of sprayed houses (stencils)					
(i) spray cycle (mentioned)	51 (100%)	0 (0.00%)	112 (100%)	0 (0.00%)	0 (0.00%)
(ii) group number (mentioned)	0 (0.00%)	0 (0.00%)	94 (83.9%)	0 (0.00%)	0 (0.00%)
(iii) team number (mentioned)	51 (100%)	0 (0.00%)	94 (83.9%)	120 (100%)	120 (100%)
(iv) spray man number (mentioned)	0 (0.00%)	0 (0.00%)	94 (83.9%)	120 (100%)	120 (100%)
(v) number or rooms sprayed	0 (0.00%)	0 (0.00%)	94 (83.9%)	0 (0.00%)	0 (0.00%)
(vi) name of the insecticide sprayed	51 (100%)	0 (0.00%)	94 (83.9%)	0 (0.00%)	0 (0.00%)
(vii) number of charges applied	0 (0.00%)	0 (0.00%)	94 (83.9%)	0 (0.00%)	0 (0.00%)
(viii) date of spray (dd/mm/yy-mentioned)	51 (100%)	0 (0.00%)	112 (100%)	120 (100%)	120 (100%)
Use of safety measures:					
(i) masks	0 (0.00%)	1 (33.3%)	112 (100%)	105 (87.5%)	111 (92.5%)
(ii) gloves	0 (0.00%)	0 (0.00%)	58 (51.8%)	0 (0.00%)	0 (0.00%)
(iii) coat/apron	0 (0.00%)	0 (0.00%)	37 (33%)	0 (0.00%)	0 (0.00%)
(iv) caps	0 (0.00%)	0 (0.00%)	51 (45.9%)	0 (0.00%)	0 (0.00%)
(v) boots	0 (0.00%)	0 (0.00%)	49 (44.1%)	0 (0.00%)	0 (0.00%)
(vi) goggles	0 (0.00%)	0 (0.00%)	10 (9%)	56 (46.7%)	111 (92.5%)
Supervisor was present during spraying	51 (100%)	0 (0.00%)	0 (0.00%)	53 (44.2%)	120 (100%)
Instruction given to the households					
(i) to stay outside during spraying	100 (100%)	31 (100%)	112 (100%)	120 (100%)	120 (100%)
(ii) to prepare the room before spraying	100 (100%)	31 (100%)	112 (100.0)	120 (100%)	120 (100%)
How were the leftover insecticide handled					
(i) buried	0 (0.00%)	0 (0.00%)	112 (100%)	43 (35.8%)	9 (7.5%)
(ii) poured into nearby water	0 (0.00%)	0 (0.00%)	0 (0.00%)	0 (0.00%)	108 (90%)
(iii) keep it for future use	0 (0.00%)	0 (0.00%)	0 (0.00%)	0 (0.00%)	0 (0.00%)
(iv) no left over	51 (100%)	31 (100%)*	0 (0.00%)	77 (64.2%)	3 (2.5%)
How insecticide pouch and sacks disposed					
(i) buried	0 (0.00%)	0 (0.00%)	112 (100%)	0 (0.00%)	0 (0.00%)
(ii) thrown nearby water	0 (0.00%)	0 (0.00%)	0 (0.00%)	0 (0.00%)	0 (0.00%)
(iii) kept for future use	0 (0.00%)	0 (0.00%)	0 (0.00%)	0 (0.00%)	0 (0.00%)
(iv) no left over	6 (1.4%)	0 (0.00%)	0 (0.00%)	0 (0.00%)	0 (0.00%)
(v) squad leader took to UHC/PHC/supervisor	45 (88.2%)	31 (100%)	0 (0.00%)	120 (100%)	120 (100%)

*Used for partial spray of unsprayed house.

**Table 3:** Findings of output indicators: review of documents of spraying programme at district/subdistrict after IRS.

Statements	Samastipur, Muzaffarpur, Vaishali (India)	Sunsari, Morang, Saptari (Nepal)	Sarlahi, Dhanusa, Mahottari (Nepal)	Fulbaria (Bangladesh)	Trishal (Bangladesh)
Record forms available (seen by observer)	Yes	Yes	Yes	Yes	Yes
Timely reporting (within 1 week of spraying)	Yes	Yes	Yes	Yes	Yes
Completeness of record (record from all the sprayed areas)	Yes	Yes	Yes	Yes	Yes
Number of targeted houses for spraying (calculate structures into houses)	1,120,946	65,30	8,289	3,032	2,833
Targeted population (the population targeted for spraying in the action plan)	5,759,799	129,550	87,570	13,611	12,822
How many squad/team you need for targeted population?	734	31	55	2	3
Spray pumps:					
(i) total pumps (district/subdistrict level)	1144	242	226	10	10
(ii) functioning	849	124	147	10	10
(iii) repairable	289	80	70	0	0
(iv) unrepairable	6	38	9	0	0
Sprayed households (% of target achieved according to spraying squads)	716,498 (63.9%)	6,290 (96.3%)	7611 (97.0% )	3,032 (100%)	2,833 (100%)
Covered population (% of population protected according to spraying squads)	3,740,157 (64.9%)	124,745 (96.3%)	74300 (95%)	13,611 (100%)	12822 (100% of target)

**Table 4:** Findings of output indicators: IRS acceptability at community level.

	Samastipur, Muzaffarpur, Vaishali (India) *N* = 419	Sunsari, Morang, *Saptari * (Nepal) *N* = 395	Sarlahi, Dhanusa, Mahottari (Nepal) *N* = 245	Fulbaria (Bangladesh) *N* = 420	Trishal (Bangladesh) *N* = 419
	*n* (%)	*n* (%)	*n* (%)	*n* (%)	*n* (%)
Do you like your house to be sprayed?	419 (100%)	363 (91.9%)	233 (95.1%)	357 (85%)	419 (100%)
After spraying do you have any side effect (multiple answer allowed)?					
(i) vomiting	0 (0.0%)	3 (0.76%)	0 (0.0%)	0 (0.0%)	0 (0.0%)
(ii) sneezing	10 (2.3%)	1 (0.25%)	0 (0.0%)	7 (1.6%)	3 (0.7%)
(iii) itching	3 (0.7%)	6 (1.52%)	0 (0.0%)	5 (1.1%)	4 (1.0%)
(iv) dizziness	0 (0.0%)	0 (0.0%)	0 (0.0%)	3 (0.7%)	0 (0.0)
(v) headache	0 (0.0%)	1 (0.25%)	0 (0.0%)	7 (1.6%)	5 (1.2%)
(vi) nausea	0 (0.0%)	1 (0.25%)	0 (0.0%)	8 (1.9%)	5 (1.2%)
(vii) others	0 (0.0%)	0 (0.0%)	0 (0.0%)	0 (0.0%)	0 (0.0%)
Before spraying did you get any advice like removing or covering (number and % of responses)?					
(i) cloths	329 (78.5%)	385 (97.5)	245 (100)	420 (100)	418 (99.5)
(ii) food/utensils	395 (94.2%)	385 (97.5)	245 (100)	420 (100)	418 (99.5)
(iii) children	419 (100%)	384 (97.2)	245 (100)	420 (100)	417 (99.3)
(iv) animals take out from cattle shed	336 (80.1%)	352 (89.11)	245 (100)	420 (100)	410 (97.6)
Have you been advised about the time you should wait to enter the house after spraying is completed?	138 (32.9%)	140 (35.4)	245 (100.0)	142 (33.8)	102 (24.3)
Have you been advised about the time you should not mud plaster or paint the wall after spraying	87 (20.7)	113 (28.6)	Not done	420 (100)	187 (44.6)

**Table 5:** Findings of outcome indicator: Sandfly density.

	Mean number *P. argentipes *			% reduction attributed*	
	Intervention	Sentinel	Control	Intervention	Sentinel
*Samastipur, Muzaffarpur, Vaishali (India)*					
(i) Baseline	2.81	2.26	2.19	—	—
(ii) 2 weeks followup	0.24	2.43	2.54	−103.91	−7.96
(iii) 4 weeks followup	0.5	2.49	2.53	−94.31	−4.87
(iv) 5 months followup	1.15	1.83	2.06	−54.45	−13.27
*Sunsari, Morang, Saptari (Nepal)*					
(i) Baseline	5.08	4.71	16.92	—	—
(ii) 2 weeks followup	0.27	0.19	15.92	−75.00	−74.73
(iii) 4 weeks followup	1.45	3.79	8.38	96.65	161.78
(iv) 5 months followup	2.21	1.56	7.67	125.59	129.51
*Fulbaria (Bangladesh)*					
(i) Baseline	13.94	—	27.49	—	—
(ii) 2 weeks followup	—	—	—	—	—
(iii) 4 weeks followup	3.53	—	11.19	42.25	—
(iv) 5 months followup	2.64	—	15.99	1.43	—
*Trishal (Bangladesh)*					
(i) Baseline	5.4	—	4.5	—	—
(ii) 2 weeks followup	—	—	—	—	—
(iii) 4 weeks followup	1.22	—	1.22	−16.67	—
(iv) 5 months followup	0.06	—	0.32	−21.48	—

*Note: negative and positive signs represent reduction and increment of *P. argentipes* after intervention, respectively.

## References

[1] Chowdhury R, Bhattacharya SK (2009). Visceral leishmaniasis: elimination from Indian sub-continent. *Proceedings of the National Academy of Sciences of India*.

[2] Bhattacharya SK, Sur D, Sinha PK, Karbwang J (2006). Elimination of leishmaniasis (kala-azar) from the Indian subcontinent is technically feasible & operationally achievable. *Indian Journal of Medical Research*.

[3] World Health Organization

[4] Mondal D, Alam MS, Karim Z, Haque R, Boelaert M, Kroeger A (2008). Present situation of vector-control management in Bangladesh: a wake up call. *Health Policy*.

[5] Joshi AB, Das ML, Akhter S (2009). Chemical and environmental vector control as a contribution to the elimination of visceral leishmaniasis on the Indian subcontinent: cluster randomized controlled trials in Bangladesh, India and Nepal. *BMC Medicine*.

[6] Chowdhury R, Huda MM, Kumar V (2011). The Indian and Nepalese programmes of indoor residual spraying for the elimination of visceral leishmaniasis: performance and effectiveness. *Annals of Tropical Medicine and Parasitology*.

[7] http://apps.who.int/tdr/svc/publications/tdr-research-publications/irs_toolkit.

[8] World Health Organization (1998). Test procedures for insecticide resistance monitoring in malaria vectors, bio-efficacy and persistence of insecticides on treated surfaces: report of the WHO informal consultation. *WHO/CDS/MAL*.

[9] World Health Organization (WHO) (2005). Guidelines for laboratory and field testing of long lasting insecticidal mosquito nets. *WHO/CDS/WHO-PES/GCDPP*.

[10] Abbott WS (1925). A method of computing the effectiveness of an insecticide. *Journal of Economic Entomology*.

[11] Dinesh DS, Das P, Picado A (2008). The efficacy of indoor CDC light traps for collecting the sandfly *Phlebotomus argentipes*, vector of *Leishmania donovani*. *Medical and Veterinary Entomology*.

[12] Lewis DJ (1982). A taxonomic review of the genus Phlebotomus (Diptera: Psychodidae). *Bulletin of the British Museum (Natural History), Entomology Series*.

[13] Mondal D, Huda MM, Karmoker MK, Ghosh D, Matlashewski G, Kroeger A Reduction of visceral Leishmaniasis by a community-based bed-net impregnation program with a slow release insecticide.

[14] Dinesh DS, Das ML, Picado A (2010). Insecticide susceptibility of Phlebotomus argentipes in visceral leishmaniasis endemic districts in India and Nepal. *PLoS Neglected Tropical Diseases*.

[15] Morou E, Ismail HM, Dowd AJ (2008). A dehydrochlorinase-based pH change assay for determination of DDT in sprayed surfaces. *Analytical Biochemistry*.

[16] Kumar V, Kesari SN, Chowdhury R User friendliness, efficiency and spray quality of stirrup pumps versus hand compression pumps for indoor residual spraying in the Visceral Leishmaniasis Elimination Programme in India.

